# CRF-Based Model for Instrument Detection and Pose Estimation in Retinal Microsurgery

**DOI:** 10.1155/2016/1067509

**Published:** 2016-10-27

**Authors:** Mohamed Alsheakhali, Abouzar Eslami, Hessam Roodaki, Nassir Navab

**Affiliations:** ^1^Technische Universität München, Munich, Germany; ^2^Carl Zeiss Meditec AG, Munich, Germany

## Abstract

Detection of instrument tip in retinal microsurgery videos is extremely challenging due to rapid motion, illumination changes, the cluttered background, and the deformable shape of the instrument. For the same reason, frequent failures in tracking add the overhead of reinitialization of the tracking. In this work, a new method is proposed to localize not only the instrument center point but also its tips and orientation without the need of manual reinitialization. Our approach models the instrument as a Conditional Random Field (CRF) where each part of the instrument is detected separately. The relations between these parts are modeled to capture the translation, rotation, and the scale changes of the instrument. The tracking is done via separate detection of instrument parts and evaluation of confidence via the modeled dependence functions. In case of low confidence feedback an automatic recovery process is performed. The algorithm is evaluated on in vivo ophthalmic surgery datasets and its performance is comparable to the state-of-the-art methods with the advantage that no manual reinitialization is needed.

## 1. Introduction

**Figure 1:**
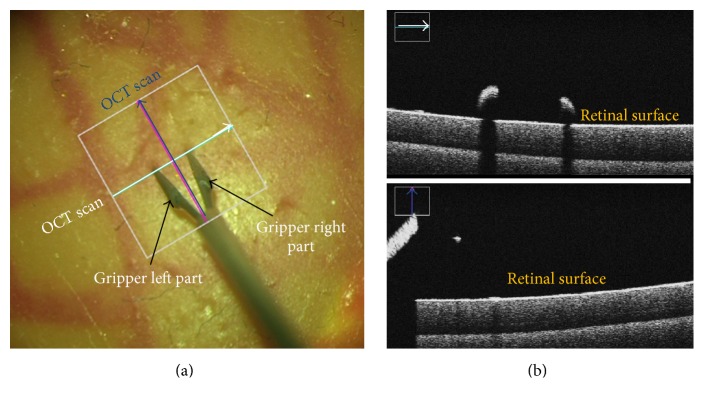
(a) Microscopic image with two OCT scans in a cross sign; (b) OCT depth information along each scan.

Retinal microsurgery is among the most delicate operations requiring microprecision medical instruments. Usage of such instruments is manually carried out by surgeons to manipulate retinal tissue. An efficient feedback of the distance between the instrument tip and the retina would minimize tissue damage caused by unintentional touch of retina. Recently, ophthalmic surgical microscopes are equipped with intraoperative Optical Coherence Tomography (OCT), which has been used in [1] to estimate the distance of the instrument to the retinal surface. However, continuous real-time detection of the instrument tip and instrument orientation is still required to enable automatic repositioning of the OCT scans during live surgery.  Figure 1 depicts two OCT scans acquired at the instrument tip and in the orientation of its shaft. The proper position is marked with a cross. The first scan (white color) is positioned at the two tips, and the corresponding OCT cross-section is shown in the upper part of Figure 1(b) which shows the retinal surface and how far the two tips are from it. The second orthogonal scan (blue color) is positioned along the instrument shaft and the cross-section corresponding to this scan is shown in the lower part of Figure 1(b). The depth information here shows the retinal surface and depth of the forceps center point. Augmenting the scene with depth information in addition to the 2D coordinates of the instruments tips brings new advantages for minimally invasive procedures. Detecting and tracking the instrument tips are needed to provide the OCT device with the new position information, and it is the most challenging step, especially for forceps instruments. Many factors such as the cluttered background, presence of blood vessels, instrument shadow, and rapid illumination have negative impact on tracking quality. Recent approaches [2, 3] modeled the instrument as a multiparts object where the parts are connected to each other in a linear way. Such approaches do not have the ability to detect the instrument tips in case of forceps usage where the linearity condition of the parts distribution is not satisfied.

In this paper, a new instrument detection, tracking, and pose estimation solution is presented. This solution relaxes the linear configuration of the instrument's parts and provides more robust model to handle different types of forceps used in eye surgery. Our method models the instrument as a Conditional Random Field (CRF) in which different parts of the instrument are detected in the 2D space of the image. Multiple models are implemented to capture the translation, rotation, and scale changes among the parts. One great advantage of the approach over the state-of-the-art methods is the ability to handle tracking failures in real-time. Such circumstances occur often in real complex datasets. The algorithm maintains confidence values to know whether to keep tracking by detection or to reinitialize the detection automatically. A second achievement of our approach is that it is the first proposed method, to the best of our knowledge, that can locate not only the instrument tips, but also its orientation in case of forceps instrument. Therefore, it provides all parameters needed to position OCT scans to get the distance between the tips and the retinal surface. Experimental results demonstrate the efficiency, robustness, and accuracy of our method in real in vivo scenarios and its ability to work on long videos. Comparisons with the state of the art on public and laparoscopic datasets demonstrate comparable results with the advantage that no manual reinitialization is needed.

## 2. Previous Work

Much research has been done to address the problem of detecting and tracking medical instruments including color-based [4, 5] and geometry-based [6–8] approaches. A recent work of Roodaki et al. [1] proposed to estimate the instrument tip depth to retina surface by building their method on top of instrument tracking algorithms. Despite of the high accuracy of the estimated depth, the algorithm relies on manual positioning of OCT scans or tracking algorithms which are prone to fail under high appearance changes. Many algorithms for instrument tracking and detection have been developed to be integrated with OCT depth estimation algorithms. However, there are many limitations of these algorithms preventing them to be used in real in vivo surgery. Sznitman et al. [9] proposed a unified framework to solve detection and tracking as a density estimation problem. The basis for this method is to model the instrument localization as a sequential entropy minimization problem to estimate 3DOF parameters required to localize the instrument tip. The method was evaluated using simple vitrectomy instrument, and it is not working on forceps used in retinal peeling operations. Therefore, such a method cannot localize the forceps two tips for automatic positioning of OCT scans. Modeling the instrument as multiple linearly connected parts was proposed in [2], but the linearity constraint limits its capabilities to detect only the center point in case of forceps instrument which is not sufficient for minimally invasive procedures. Machine learning based detectors [10] and online learning methods [11] have been employed to track only the center point of the instrument without detecting forceps tips. Reiter et al. [6] proposed a solution to track the instrument by making use of the landmarks on its surface. Color, location, and gradient-based features have been associated with the landmarks for training random ferns. The 3D locations of the instrument are retrieved by matching the features tracks in the stereo camera using normalized cross correlation. The method achieves high localization accuracy. However, it cannot run at the video frame rate due to the computational cost of extracting all these features. Moreover, the occlusions of some landmarks due to the instrument rotation might result in high localization error. Another approach [12] was proposed for articulated instrument tracking in 3D laparoscopic images, in which the color information is used for instrument parts segmentation. The segmented regions are described by different statistical models in order to estimate the pose of the instrument in the 3D space. Optical flow is used for pose tracking from image to another. The approach has also the limitations of expensive feature extraction and high sensitivity to the light changes. Rieke et al. [13] proposed to use regression forests to localize the forceps tips within a bounding box. However, this bounding box is provided using intensity-based tracker. Hence, once the tracker gets lost, the operation has to be interrupted to reinitialize OCT device manually. A recent work [3] proposed to use the deep learning to detect the instrument parts and estimate its orientation. The approach achieved comparable results to the state-of-the-art methods but it is computationally expensive as well as it cannot detect the two forceps tips. Generally, most of the limitations are due to the time complexity or inability to detect forceps two tips and forceps orientation which are addressed in the work of this paper.

## 3. Proposed Method

**Figure 2:**
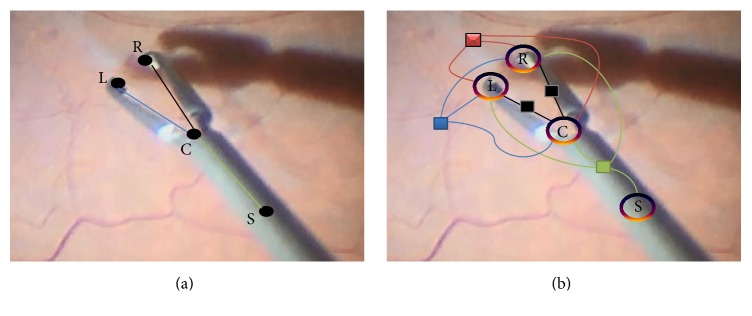
(a) Target pose estimation; (b) the factor graph for the forceps: 4 variables (left (L), right (R), center (C), and shaft (S)) are used with different types of constraints that are presented with different edge colors: black (translation), green (rotation), red (relative length), and blue (consistency).

Medical instrument, in this work, is modeled as multiparts articulated object where each part can be detected separately. Depending on the used features, parts detections using most of machine learning classifiers can result in a large number of false detections especially for structure-less objects like our target. However, these detections, including the true positive ones, form a new and reduced search space within the 2D image space which represents instrument part's hypotheses space. Therefore, the sought targets are just specific instrument part detections within the reduced space, such that the detected parts would represent the final instrument configuration. Prior information about the instrument parts and the relations between them are integrated on top of these detections together in one model in order to filter out the vast majority of false detections and to end up with the optimal instrument configuration. Prior instrument information can include the relative lengths of the parts, the angles between them, the gripper length, the possible movements of the joint, the possible changes of the current state, and so forth. Given different models, expressed as probabilistic distributions, to describe prior information about the instrument, and some potential instrument configurations, then the ultimate goal of our approach is to optimize for the best configuration (instrument pose) as shown in Figure 2(a) which maximizes the likelihood of the distributions of the prior models. To this end, the instrument in our method is modeled as a CRF of *n* random variables, and the factor graph of this model is shown in Figure 2(b). Each random variable *Y*_*i*_ corresponds to an instrument part, and the edges among these variables denote conditional dependence of the parts which can be described as a physical constraint. The instrument pose is given by the configuration *Y* = (*Y*_1_, *Y*_2_,…, *Y*_*n*_), where the state for each variable *Y*_*i*_ ∈ Λ_*i*_ represents the 2D position of the instrument part and is taken from the discrete space Λ_*i*_ ⊂ *R* ^2^. Consider an instance of the observation *x* ∈ *X* that corresponds to instrument parts features, a reference pose *P*, and an instrument configuration *y* ∈ *Y*; the posterior is defined as

(1) py ∣ x,P=1Zx,P∏inΦiConfyi,x·ΦiTempyi,Pi·∏i,j∈ETransΨConnyi,yj·∏i,j,k∈ERLenΨRLenyi,yj,yk·∏i,j,k∈EConsΨConsyi,yj,yk·∏i,j,k,l∈ERotΨRotyi,yj,yk,yl,

where *Z*(*x*, *P*) is the partition function and Φ^Conf^(*y*_*i*_, *x*) is the unary score function. *E*_Trans_, *E*_RLen_, *E*_Cons_, and *E*_Rot_ are the graph edges that model the kinematic constraints among the instrument parts using different potentials functions. Ψ^Conn^ is binary potentials functions to model the distances changes among the forceps gripper's end points based on the connectivity between the forceps center point and each of the tips. Ψ^RLen^ and Ψ^Cons^ are ternary potentials functions to ensure consistency in the relative length of the left and right parts of the gripper and whether they can be bounded by a small region in the image. The rotation potential function Ψ^Rot^ is defined to estimate the configuration likelihood based on the distribution describing the proper angles among the instrument parts. Once the forceps hypothetical parts are detected, different configurations from these hypotheses within a defined Region of Interest (ROI) are evaluated with the potential functions to select one configuration. This configuration is the one maximizing the posterior given in (1) and it represents the forceps pose.

In the next sections, we present the unary potential which is used to define some probable coordinates for instrument parts, followed by different types of potential functions to impose kinematic constraints on the instrument parts and represent our prior model of the instrument.

### 3.1. Unary Potentials

The unary potential functions are designed to give a score for each instrument part hypothesis. Each hypothesis has a confidence value which is a probability assigned to the pixel in 2D images to express its degree of belonging to a specific instrument part. A regression forest [14] is trained on histogram of oriented gradients (HOG) [15] features for this purpose and regarded as a multiclass detector. The output of the regression forest is a class label prediction for each hypothesis and a confidence value. The number of class labels is set to the number of random variables in the CRF plus one for the background. The confidence value for each instrument part hypothesis is defined in

(2) $$\begin{array}{c} \Phi^{Conf}\left(\;y_{i},x\;\right)=\frac{1}{T}\overset{T}{\underset{j=1}{\sum }}\pi_{j}\left(\;x\;\right), \end{array}$$

where *T* is the number of trees in the forest and *π*_*j*_(*x*) is the probability assigned by one tree to *y*_*i*_ to express its belonging to a specific instrument part. The probability is given based on testing the features *x* associated with *y*_*i*_. The term Φ^Temp^(*y*_*i*_, *P*_*i*_) favors part hypotheses which are close to the last inferred part *P*_*i*_ based on the distance between them, as given by

(3) $$\begin{array}{c} \Phi^{Temp}\left(\;y_{i},P_{i}\;\right)=e^{-{\left\|y_{i}-P_{i}\right\|}_{2}^{2}/2}. \end{array}$$

### 3.2. Binary Translation Potentials

**Figure 3:**
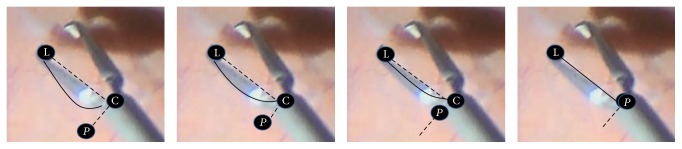
Connectivity modeling using Bézier curves where the dashed lines are orthogonal vectors and the position of the control point *p* is placed along one of those vectors with different displacements Δ*p* from the center point.

The distance between the tips and the center point changes at different scales and orientations. The translation potentials model these translations of the left and the right tips to the center point by measuring the connectivity between the hypotheses of the instrument parts involved in the translational edges as shown in Figure 2(b). For example, given one hypothesis *y*_*i*_ of the left part and one hypothesis *y*_*j*_ of the center part detections, the connectivity between them is computed along different quadratic Bézier curves controlled by the position of the control point *P* ∈ *R* ^2^, as shown in Figure 3. The control point *P* is placed along the orthogonal vector to the vector (*y*_*i*_, *y*_*j*_). The distance of the point *P* to *y*_*j*_ specifies the shape of the curve connecting *y*_*i*_ and *y*_*j*_. By denoting this curve as *C*_*y*_*i*__^*y*_*j*_^(*P*), the probabilistic connectivity along each curve is given by the following equation:

(4) $$\begin{array}{c} Conn\left(\;C_{y_{i}}^{y_{j}}\left(\;P\;\right)\;\right)=\frac{1}{k^{2}}\overset{S}{\underset{j=1}{\sum }}{\left|\;s_{j}\;\right|}^{2}, \end{array}$$

in which *k* is a normalization factor. The curve is assumed to consist of *S* ∈ *R* segments. Each segment *s*_*j*_ is a connected component of pixels along one curve. The connected components are extracted from the binary image created by thresholding the gradient image of the input microscopic image. The points *y*_*i*_ and *y*_*j*_ are overlaid on the binary image and considered strongly connected if at least one of Bézier curves aligned to the gripper edges curvature. This curve might consist of zero (not connected hypotheses where *C*_*y*_*i*__^*y*_*j*_^(*P*) is set to *ε* for numerical stability), one, or many segments. Changing the position of *P* by different Δ*p* values enables the algorithm to handle various types of forceps with different curvatures along the gripper as shown in Figure 3. The connectivity measure in (4) is modeled to favor longer segments and penalize short ones in order to be robust in case of noisy images. The translation potential function keeps the maximum probability among all curves and it is defined in (5). A higher value of this probability means stronger connectivity and higher potential of the hypotheses to belong to the gripper end points:

(5) $$\begin{array}{c} \Psi^{Conn}\left(\;y_{i},y_{j}\;\right)=\underset{\Delta p}{\max\;}Conn\left(\;C_{y_{i}}^{y_{j}}\left(\;P+\Delta p\;\right)\;\right). \end{array}$$

The connectivity along the left and right parts of the gripper are calculated in the same way but with different positioning of the control point *P*.

### 3.3. Ternary Potentials

The relative length function Ψ^RLen^ is used to model the relative length between the left and right gripper parts as a Gaussian distribution and is given in (6). The function is designed to increase the algorithm robustness in case of false detections of structures like vessels near the instrument tips. The model parameters *μ*_*i*,*j*,*k*_^RLen^ and *σ*_*i*,*j*,*k*_^RLen^ are estimated from the ground truth. Moreover, the gripper length should be consistent with shaft length in the ROI from which the configurations are selected. Hence, the consistency function Ψ^Cons^ ∈ {1, *ε*} is modeled to favor selected gripper parts with lengths less than half the size of the ROI side length. Otherwise, the output of the function is a small probability (*ε*) to penalize this configuration. In this way, the inconsistent combinations of parts hypotheses are penalized:

(6) ΨRLenyi,yj,yk=Nyi−yj,yi−ykμi,j,kRLen,σi,j,kRLen,

where *y*_*i*_, *y*_*j*_, and *y*_*k*_ are center, left, and right hypotheses, respectively.

### 3.4. Quaternary Rotation Potential

Any configuration *y* of the instrument forms an angles triple *θ* = {*θ*_*i*_, *i* = 1,2, 3} among its parts treated as random variables. The rotation potential in (7) models the relations between these random variables as a mixture of two multivariate Gaussian distributions. One distribution models the relation among the variables when the instrument is closed or is about to be closed, while the other distribution is for the open instrument with different degrees. The parameters for each distribution (the mean *μ*_*i*,*j*,*k*,*l*_^*R*_*n*_^ and the covariance Σ_*i*,*j*,*k*,*l*_^*R*_*n*_^) are estimated from the ground truth, where *n* = 1 for one distribution and *n* = 2 for the other:

(7) $$\begin{array}{c} \Psi^{Rot}\left(\;y_{i},y_{j},y_{k},y_{l}\;\right)=\overset{2}{\underset{n=1}{\sum }}N\left(\;{\left(\;\theta \;\right)}_{i,j,k,l}\;|\;\mu_{i,j,k,l}^{R_{n}},\Sigma_{i,j,k,l}^{R_{n}}\;\right), \end{array}$$

where *y*_*i*_, *y*_*j*_, *y*_*k*_, and *y*_*l*_ are left, center, right, and shaft hypotheses, respectively.

### 3.5. Inference of the Instrument Pose

We used genetic algorithms [16] to infer an approximate solution which maximizes the posterior equation as

(8) $$\begin{array}{c} \hat{y}=\underset{y}{\arg\max\;}p\left(\;y\;|\;x,P\;\right). \end{array}$$

The most important parts of the genetic algorithms are the representation of the chromosomes and the definition of the fitness function. Each chromosome is represented by one configuration with four genes 〈*y*_*i*_, *y*_*j*_, *y*_*k*_, *y*_*l*_〉 representing the joints coordinates. The fitness function is set to the posterior function given in (1), which depends on the prior models *p*(*y*) of the instrument kinematics and the initial hypotheses probabilities given by the regression forest. The algorithm starts by initial random generation of 1000 configurations which considered the initial population. Among those configurations, the crossover is applied pairwise by interleaving the genes at specific index to generate more variations from the current population. However, to enable the algorithm skipping local maxima during optimization, mutation operation is employed to replace random genes with others from the neighborhood. The produced configurations are evaluated using the fitness function, and a new generation is formed from the best evaluated configurations. The solution is obtained after a fixed number of iterations or no convergence in two successive generations.

Once the pose is estimated in the first frame, a reduced Region of Interest (ROI) is defined around the instrument center point to limit our detection space in the next frames. This ROI is expanded gradually when any instrument part is missing in the unary detections or when the confidence from the inferred pose is low. Low confidence of the final solution after optimization happens with either (1) low likelihood of the rotation distributions or (2) the consistency potential output being small (*ε*). These cases mean either that the solution cannot have the normal forceps shape or that it has been formed from false detections in ROI, which requires the reinitialization to be triggered automatically by expanding the ROI.

## 4. Experiments and Results

The experimental validation of the proposed method is carried out on three different microsurgery datasets. The first dataset, referred to as “Zeiss dataset,” consists of eight sequences of surgeries performed on human eyes with frame resolution of 1920 × 1080 pixels, downsampled to one-fourth of the original size. Experiments on original size sequences prove the downsampling to have minimal effect on the detection accuracy. The second dataset is publicly available [10] with 1171 images of 640 × 480 pixels. No downsampling is performed on this dataset. The third dataset is a laparoscopic surgery dataset with 1000 images available on YouTube (http://www.youtube.com/watch?v=IVp1sgjQ5To). The proposed algorithm is evaluated by estimating the pose of one of the instruments present in the laparoscopic surgery since the other instrument has a fixed pose. The performance of the algorithm was evaluated using three different metrics: (1) accuracy threshold score defined by Sznitman et al. [10] to measure the pixel-wise detection accuracy for each instrument joint, (2) the strict Percentage of Correct Parts (strict PCP) [17] for gripper parts detection accuracy, and (3) the angular threshold score defined in [5] to measure the accuracy of estimating the shaft's orientation. The algorithm runs at 15-fps for public and laparoscopic datasets and 18-fps for Zeiss dataset on a normal personal computer. For the regression forest 50 trees with maximum depth of 25 are used. The HOG features bin size is set to 9 and the patch size is 50 × 50 pixels.

### 4.1. Zeiss Dataset

**Figure 4:**
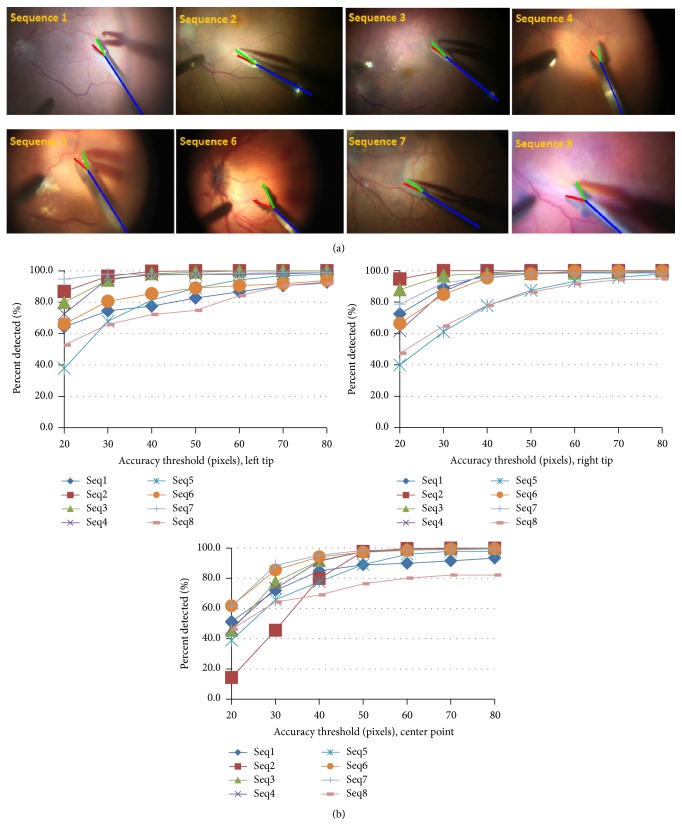
(a)  8 samples from each sequence of Zeiss dataset with pose estimation; (b) the accuracy threshold for left, right, and center points, respectively.

The algorithm was evaluated on 8 sequences as shown in Figure 4, where each sequence was taken from different surgery with different conditions. To achieve maximum reliability in clinical use, only 200 images from the first 4 sequences were used for training. The testing was done on the remaining images from each sequence in addition to 4 other unseen sequences. The number of testing images from each dataset is listed in Table 1. Each training frame has 4 annotated points: left and right tips, center point, and a point on the shaft centerline. 200 samples from the training images were manually clustered to open and close states to estimate the parameters of the rotation Gaussian distributions. Since the instrument shaft diameter is 50 pixels, we evaluate using values between 20 and 80 pixels for the accuracy threshold. Figure 4 shows the percentage of correctly predicted locations for different joints of the instrument. The results show that in 90% of the testing images the tips are detected with less than 50 pixels (the shaft diameter) error. The strict PCP scores of the left and right gripper's parts for *α* = 0.5 (which used for human pose estimation evaluation) for each sequence are depicted in Table 1 which show the robustness of the algorithm and its ability to generalize to new sequences.

### 4.2. Public Dataset

**Figure 5:**
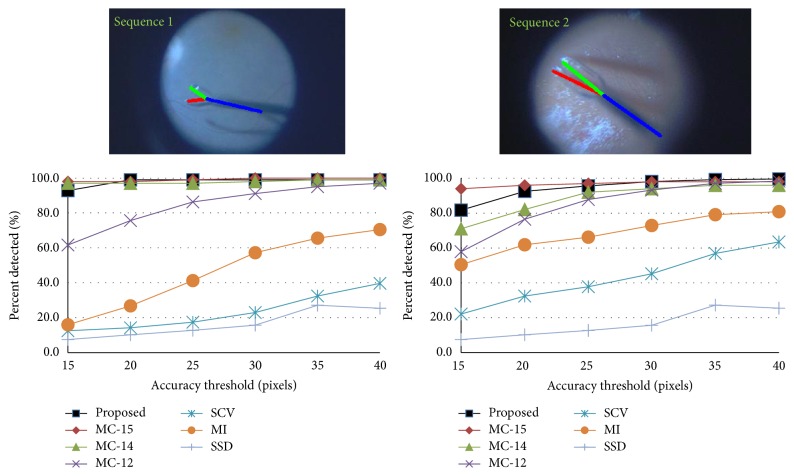
Threshold accuracy for each of the public sequences separately.

The proposed method was compared with the state-of-the art methods: MC-15 [13], MC-14 [2], MC-12 [10], SCV [18], MI [19], and SSD. The evaluation includes two sequences of the public datasets. The third sequence is omitted, as in [2], due to its short length which makes it ill-suited for training purposes. In the first experiment, the training is done separately on the first half of each sequence and testing was on the second half. The detection accuracy of the center point is shown in Figure 5 which shows comparability of the proposed method to the state-of-the-art methods with the advantage of not requiring manual reinitialization. For example, at threshold of 20 pixels (the shaft diameter), the center points are detected correctly in more than 95% of the images in both cases. The accuracy threshold scores for detecting the two tips of the forceps in each sequence are depicted in Figure 7.

In the second experiment, the training is performed on the full dataset (the first two halves of the two sequences together) and the testing is done on the second halves. The performance of detecting forceps tips and forceps center point is shown in Figure 7 labeled with the prefix full. The strict PCP scores for both experiments are listed in Table 2 and compared to MC-15 [13] which is the only state-of-the-art method that can locate the forceps tips even though it is only tracking method and uses manual initialization to handle tracking failures in live surgery.

### 4.3. Laparoscopic Dataset

**Figure 6:**
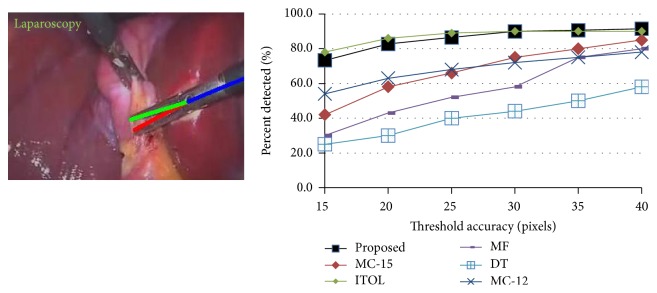
Threshold accuracy for laparoscopic dataset.

**Figure 7:**
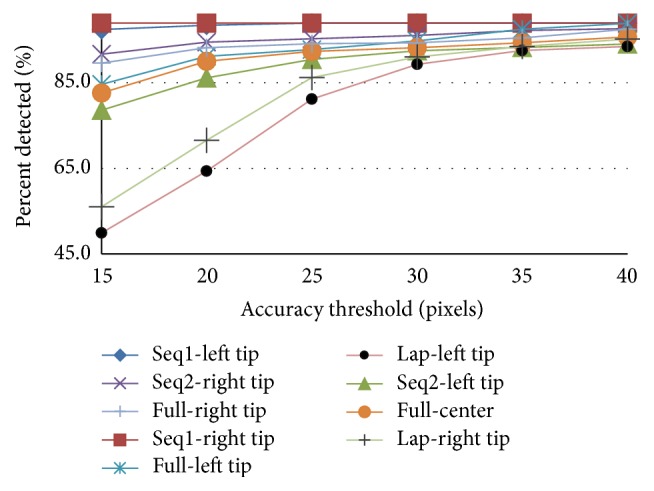
Accuracy threshold for different forceps joints of the public (full and separate sequences) and laparoscopic (Lap) datasets.

We compared our performance with MC-15 [13], MC-12 [10], ITOL [11], MF [11], and DT [11]. Similar to these methods, training was done on the first half of the dataset and the testing on the second half. Comparing the performance of our method in detecting the center point with the other methods using accuracy threshold is shown in Figure 6. It is obvious that our method outperforms most state-of-the-art methods and achieves similar results to ITOL which is also a tracking method and impractical for live surgery due to the required manual reinitialization. The accuracy threshold scores of detecting each tip are shown in Figure 7 while all other methods do not detect them in this challenging dataset. The PCP scores are given in Table 2 which show even high detection accuracy of both gripper's parts.

**Figure 8:**
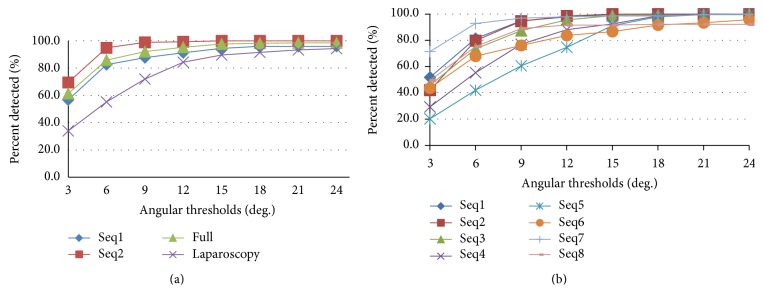
(a) Angular threshold scores for public and laparoscopic sequences; (b) angular threshold scores for Zeiss sequences.

Figures 8(a) and 8(b) show the performance of our algorithm to estimate the orientation of the shaft while varying the angular threshold from 3 to 24 deg. It is evident that in 85 percent of the images, the orientation is detected with deviation less than 15 deg.

## 5. Results Discussion

The proposed approach shows high accuracy of instrument joints localization in real-time performance. This accuracy is attributed to modeling the dependencies between instrument parts as CRF model, while other methods do not consider these dependencies and rely only on individual parts detection. These dependencies are built on top of random forest outputs trained using only gradient-based (HOG) features to serve as unary detections functions. Unlike other intensity-based tracker methods, relying on HOG features makes our approach robust enough to illumination changes during surgery. Moreover, it reduces the amount of training samples needed for training large changes in instrument appearance. This is why, in the first dataset, our algorithm needs only 200 samples from only 4 sequence and it is able to run on testing images with 3 times the size of the training ones. Practically, it can run on even longer sequences since there is no need to train more samples to account for new illumination changes. This has been proven by running the algorithm on 4 other unseen sequences and achieving high performance which is considered a great achievement of our approach in comparison with the state-of-the-art methods MC-15 [13] and ITOL [11]. Moreover, relying on detected structural parts using HOG features bring new advantage to our method which is being able to sense some confidence signals. This feedback is employed for automatic recovery process, which is missing in most other methods, to localize again the instrument after its disappearance without surgeon's intervention. The results also presented high PCP scores on most of the retinal sequences. However, in sequence 8, the PCP score is not as high as the other sequences due to the blurriness of the images which makes the detection of the gripper edges very difficult. Hence, the connectivity potential function will not be able to give fair preferences to some configurations. Coming to the public dataset, PCP scores of our method show comparable results to MC-15 [13]. However, the advantage of our approach is the ability to work without stopping on these sequences, while in sequence 2, MC-15 [13] needed the manual reinitialization twice to handle instrument disappearance from the scene. Comparing on laparoscopic dataset, our approach outperforms MC-15 [13] by at least 20% at most of the accuracy thresholds in localizing the instrument center point as shown in Figure 6 and achieves very close performance to ITOL [11]. However, ITOL cannot detect the forceps two tips as well as it is just intensity-based tracking algorithm. Hence, our algorithm tends to be more robust and practical for real surgeries due to its ability to localize the instrument left and right tips with high accuracy.

One more important strength point of the proposed approach is the ability to estimate the orientation of the instrument shaft. Unlike other approaches, the orientation is treated as a part in our CRF model, and this characteristic makes our approach successful one for the full integration with OCT imaging to position OCT scans according to given coordinates and orientation. The angular threshold results show also high accuracy in estimating the instrument orientation in all sequences of the different datasets.

## 6. Conclusions

We presented a new approach for localizing the forceps tips and center point as well as estimating the orientation of its shaft. The approach models the instrument detection, tracking, and pose estimation as a CRF's inference problem. The performance of the proposed approach has been evaluated on retinal and laparoscopic surgeries using three different metrics. The algorithm generates all parameters needed for OCT device in order to position OCT scans automatically in real surgery. It also achieves real-time performance and works on real surgery sequences. Moreover, it does not require manual initialization since it tracks the instrument by constantly detecting its parts and maintains a confidence value to reinitialize the detection automatically whenever it is needed. The method demonstrates high detection rate of the instrument joints on long sequences as well as comparable results to the state-of-the-art methods without the need of manual reinitialization.

## Figures and Tables

**Table 1:** Strict PCP scores for *α* = 0.5 on Zeiss dataset.

Zeiss sequences	Seq 1	Seq 2	Seq 3	Seq 4	Seq 5	Seq 6	Seq 7	Seq 8
#Testing images	590	400	400	400	200	400	200	200

Left PCP	91	99	98	98	92	85	96	75
Right PCP	93	99	99	99	93	94	97	76

**Table 2:** Strict PCP scores for *α* = 0.5 on public and laparoscopic (Lap) datasets.

	Proposed				MC-15			
Public/Lap sequences	Seq 1	Seq 2	Full	Lap	Seq 1	Seq 2	Full	Lap
Left PCP	**97**	93	89	89	95	**97**	N/A	N/A
Right PCP	95	** 95**	89	90	** 97 **	** 95**	N/A	N/A
